# Understanding the Factors Explaining the Growing Use of Medical Assistance in Dying in Québec: Protocol for an Interdisciplinary Mixed Methods and Multimethods Study

**DOI:** 10.2196/83549

**Published:** 2026-04-20

**Authors:** Marie-Ève Bouthillier, Isabelle Marcoux, Catherine Perron, Bruno Gagnon, David Lussier, Ghislaine Rouly, Mathieu Moreau, Michel Dorval, Sabrina Lessard, Dominique Girard, Gina Bravo, Maude Hébert, Michelle Giroux, Simon Lemyre, Alexandra Beaudin, Claude Julie Bourque, Maryse Soulières, Maude Lévesque, Mona Gupta, Valérie Bourgeois-Guérin, David Lavoie, Bertrand Lavoie, Ariane Plaisance, Louise Bernier, Jacinthe Dupuis

**Affiliations:** 1 Department of Family Medicine and Emergency Medicine Faculty of Medicine Université de Montréal Montréal, QC Canada; 2 Research Centre Centre Hospitalier de l’Université de Montréal Montréal, QC Canada; 3 Interdisciplinary School of Health Sciences Faculty of Health Sciences University of Ottawa Ottawa, ON Canada; 4 School of Social Work Faculty of Arts and Humanities Université de Sherbrooke Sherbrooke, QC Canada; 5 Department of Family Medicine and Emergency Medicine Centre de recherche du CHU de Québec-Université Laval Québec, QC Canada; 6 VITAM Sustainable Health Research Center Université Laval Québec, QC Canada; 7 Department of Medicine Faculty of Medicine Université de Montréal Montréal, QC Canada; 8 Faculty of Pharmacy Université Laval Québec, QC Canada; 9 Centre de recherche du CHU de Québec-Université Laval Québec, QC Canada; 10 Department of Anthropology Faculty of Arts and Sciences Université de Montréal Montréal, QC Canada; 11 Centre for Research and Expertise in Social Gerontology Montréal, QC Canada; 12 Department of Health Sciences Université du Québec à Rimouski Rimouski, QC Canada; 13 Department of Community Health Sciences Faculty of Medicine and Health Sciences Université de Sherbrooke Sherbrooke, QC Canada; 14 Department of Nursing Université du Québec à Trois-Rivières Trois-Rivières, QC Canada; 15 Civil Law Section Law Faculty University of Ottawa Ottawa, ON Canada; 16 Centre de recherche en pédagogie de la santé Faculté de médecine Université de Montréal Montréal, QC Canada; 17 School of Social Work Université de Montréal Montréal, QC Canada; 18 School of Social Work Université du Québec à Montréal Montréal, QC Canada; 19 Department of Psychiatry and Addiction Medicine Faculty of Medicine Université de Montréal Montréal, QC Canada; 20 Department of Psychology Université du Québec à Montréal Montréal, QC Canada; 21 Law Faculty Université de Sherbrooke Sherbrooke, QC Canada; 22 Qulysis Québec, QC Canada; 23 Centre Integre de Sante et de Services Sociaux de Laval Laval, QC Canada

**Keywords:** assisted dying related factors, Canada, end-of-life care, euthanasia, evolution of assisted dying practices, factors explaining use of MAiD, growing use of MAiD, medical assistance in dying, mixed and multimethod study, protocol, Québec

## Abstract

**Background:**

Medical assistance in dying (MAiD) became a legal end-of-life option on December 10, 2015, in Québec, and on June 17, 2016, in the rest of Canada. Since its legalization, there has been a steady increase in the number of MAiD requests and provisions. Across permissive jurisdictions, Québec now has the highest rate of assisted death. Despite the growing use of MAiD, research examining the factors driving this increase remains limited and fragmented. Existing studies offer partial and sometimes contradictory explanations, with little integration of legal, institutional, societal, and individual dimensions. Further research is needed to better understand the determinants of MAiD requests and practices, particularly in the Canadian and Québec contexts.

**Objective:**

This research aims to understand the factors influencing changes in MAiD requests and administrations in Québec by examining laws, practices, societal perspectives, organization of care and services, and individual characteristics of those requesting MAiD, as well as their interrelationships. We present the protocol developed by the Consortium interdisciplinaire de recherche sur l'aide médicale à mourir, an interdisciplinary research consortium, including an international advisory committee, set up for this research.

**Methods:**

The design of this protocol is multimethods and convergent mixed methods, including (1) an international cross-thematical approach with 4 main research methods (a scoping review, key informant interviews, focus groups with health care professionals, and a population-based survey) chosen to partially answer research questions across the entire study and to compare with other jurisdictions and (2) 11 theme-specific methods (including community forums, media coverage analysis, comparative legal analyses, case studies of triads, individual interviews, and system mapping) to enrich and complement findings from the cross-thematical approach.

**Results:**

When this 3-year funded study started in July 2024, several research methods not requiring ethics committee approval (because no human participants were involved) were initiated, including scoping and systematic reviews, media coverage analysis, and comparative legal analyses. By August 2025, interviews with key informants were completed, and analyses took place in September. Concurrently, other subteams started data collection (focus groups December 2025) or are getting ready to seek ethics approval for their protocols and data collection processes involving human participants: case studies of triads, individual interviews, and community forums.

**Conclusions:**

Findings from the international cross-thematical approach and theme-specific methods will provide a comprehensive understanding of the factors influencing the use of MAiD in Québec. This study has strengths, including the use of a specific theoretical framework, a variety of complementary methods, and an integrated knowledge mobilization strategy. As for its limitations, we foresee challenges with the comparison of jurisdictions in terms of language, culture, and legal systems, as well as access to data about MAiD cases, since reporting systems may differ between jurisdictions.

**International Registered Report Identifier (IRRID):**

DERR1-10.2196/83549

## Introduction

### Overview

In the province of Québec, medical assistance in dying (MAiD) has been permitted, as of December 2015, for individuals who meet the eligibility criteria specified in the Act respecting end-of-life care [1]. This legislation sets out a framework for “end-of-life care,” which includes palliative care, continuous palliative sedation, as well as euthanasia (referred to in the Act and in this paper as MAiD) [1]. In Québec, MAiD consists “in the administration by a competent professional of medications or substances to a patient, at the patient’s request, in order to relieve their suffering by hastening death.” [2]. Unlike the Canadian law, Québec legislation excludes assisted suicide or self-administered MAiD [3].

Since 2015, there has been a steady year-over-year increase in the global number of requests and provisions of assisted dying [4]. In fact, Québec now ranks first in the world in terms of the proportion of MAiD deaths, ahead of other Canadian provinces, and even of countries where this practice has been permitted for decades [5,6]. In the September of 2023, at the request of the Québec Ministry of Health and Social Services, a call for proposals was launched by Québec’s main funding agency (Fonds de recherche du Québec) to fund a research team willing to investigate the factors explaining this rise in MAiD incidence [7]. The study proposed by our consortium (Consortium interdisciplinaire de recherche sur l'aide médicale à mourir [CIRAMM]; in English: Interdisciplinary Research Consortium on Medical Assistance in Dying) was successful in securing 3-year financial support [8]. The objectives of this paper are the following: (1) to briefly summarize current knowledge on the factors explaining the use of MAiD in Québec, (2) to specify the research objectives that led to the development of the CIRAMM to carry out this vast study, (3) to present the study’s theoretical framework, (4) to describe its methodology, and (5) to discuss its strengths and limitations.

### Factors Explaining the Use of MAiD

Studies on the evolution of the use of MAiD and potential explanatory factors remain sparse. Few authors propose hypotheses concerning the increase in MAiD practices at the international level. Boer [9] describes the development of medically assisted death in the Netherlands from 1968 onward, and reports that the number of euthanasia tripled between 2007 and 2021; a period during which eligibility criteria would have been interpreted more broadly, and mobile teams performing euthanasia without a prior doctor-patient relationship were set up. Still in the Netherlands, a study conducted by Groenewoud et al [10] revealed that age, church attendance, political orientation, income, health, and volunteer availability were associated with the geographical variation in the incidence of euthanasia over time (2013-2017).

Byram and Reiner [11] tested 10 hypotheses that could explain why the rates of deaths by MAiD are 15 times higher in Canada than those by assisted suicide in California, despite having adopted their respective laws in 2016. Three of them were confirmed: (1) limited public awareness, (2) fewer MAiD practitioners per capita, and (3) sparse support by health care institutions in California in comparison to Canada. On the other hand, Pullman [12] suggests, as possible hypotheses for the higher rate of MAiD in Canada, that the eligibility criteria are more ambiguous, their interpretation more flexible, and the method used (mainly injection in Canada rather than prescription of lethal drugs in California) implies more active participation of providers, who (even if that’s not their intention), have tacit ascendancy over patients in vulnerable positions. According to Khakzedeh [13], other factors associated with the legislation and its implementation should be taken into account. For example, in jurisdictions like the United States, Switzerland, and Austria, assisted suicide is neither a care nor a right, and involves greater mobilization on the part of individuals regarding medical consultations, the purchase of medication, or notarial deeds [13].

In the Canadian context, recent analyses and opinion pieces on what triggers requests for MAiD bring divergent and controversial elements to the conversation. Coelho et al [14] link MAiD requests to suffering and lack of medical, psychosocial, and rehabilitation support (disability support) due in part to structural problems. The lack of access to palliative care and housing alternatives is often cited in the media to explain the use of MAiD [15-19]. Alternatively, according to Downar et al [20], available evidence showed that people who died by MAiD had higher socioeconomic status, and most of them benefited from the involvement of palliative care providers. In Québec, the idealization of “a good death,” social secularization, and the widespread emphasis on self-determination have been proposed to explain the increase in the use of MAiD [21,22]. The “quest for autonomy” and the reappropriation of power in the intimate sphere could be explained by the retreat (or rejection) of the argument of authority [22] and, in Québec in particular, the decline of religion [23]. Even with “increasing social acceptance of this care” [24] in Québec, as elsewhere, studies highlight the lack of public knowledge of MAiD in relation to other end-of-life practices [25,26]. Overall, the practice of euthanasia and assisted suicide is still relatively recent in Canada. Studies are needed to better understand the use of these means to end one’s life.

### Research Objectives

The call for proposals issued by the Fonds de recherche du Québec aimed at better understanding the use of MAiD in the Québec context. It included a series of stated objectives reproduced in Textbox 1.

**Research themes and objectives.**

**Personal characteristics**

- Identify the characteristics of people who request medical assistance in dying (MAiD)
- Identify how social determinants of health influence the personal experience of MAiD
- Investigate the influence of vulnerability factors on personal experience
- Investigate the relationship between the nature of suffering and one’s desire to obtain MAiD
- Investigate how personal experiences of support throughout the MAiD process influence one’s motivation to consider MAiD

**Organization and delivery of health care in Québec**

- Demonstrate how Québec’s end-of-life care legislative provisions that pertain to the organization of health care are associated with the increased use of MAiD
- Explain how differences in the availability of end-of-life care in different regions and institutions influence the use of MAiD
- Describe the relationship between professional practice in MAiD and the evolution of requests for MAiD in Québec

**Societal aspects**

- Determine whether sociolegal factors explain the growing use of MAiD
- Examine whether the patient (family)/professional relationship has an impact on MAiD requests and administration
- Explore the factors influencing the social acceptability of MAiD
- Understand local and regional disparities in MAiD with respect to their sociodemographic characteristics

**Practices, legislation, and public policies**

- Compare and contrast Québec’s MAiD legislation and public policies with those of other jurisdictions
- Examine how MAiD numbers in Québec compare to those in other jurisdictions
- Explore how Québec’s context can explain differences in MAiD use

**Regulation of advance requests for MAiD**

- Compare the provisions for advance MAiD requests set out in Québec’s Act respecting end-of-life-care with those in effect in jurisdictions that already allow MAiD for incompetent people
- Document experiences and practices related to advance MAiD requests in these other jurisdictions

### Theoretical Framework

**Figure 1:**
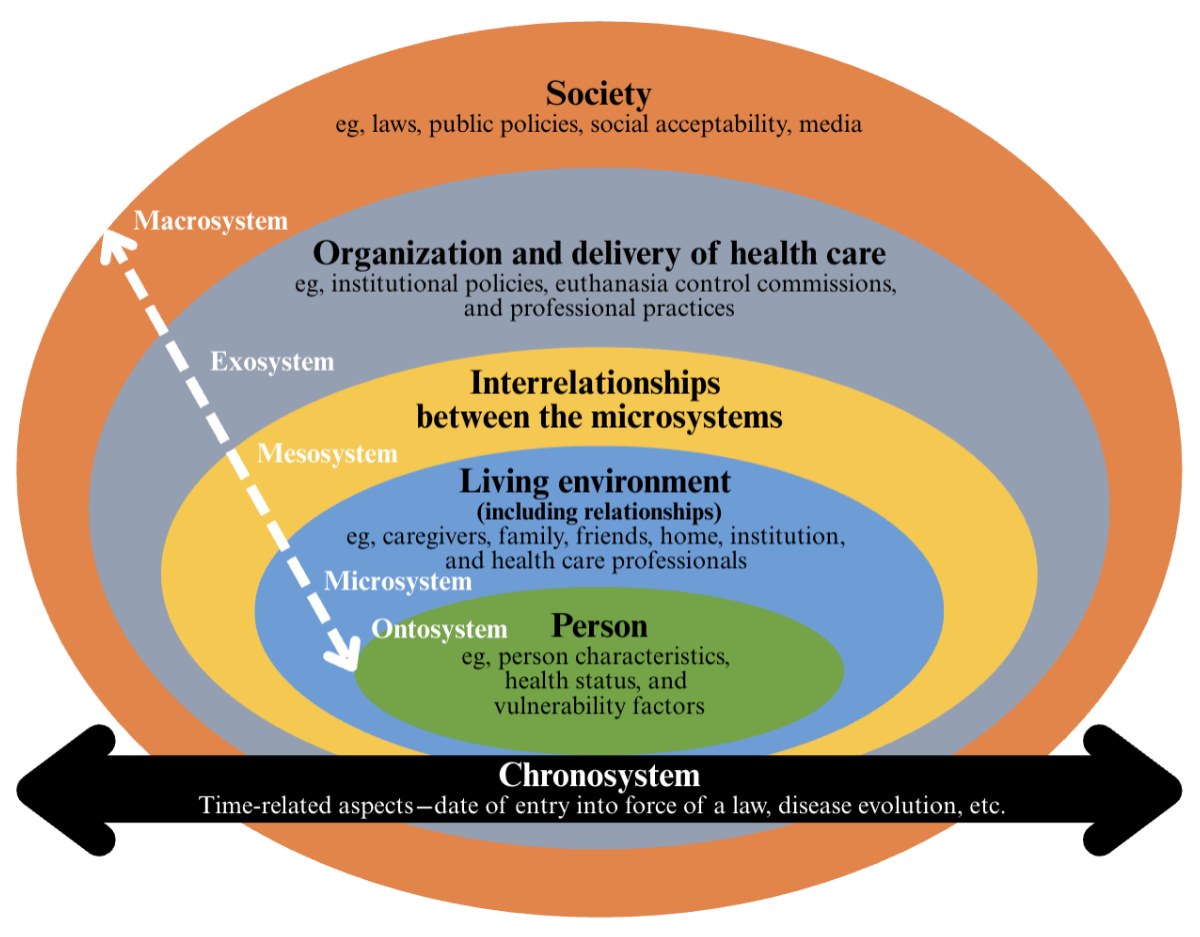
Bronfenbrenner ecological systems theory to better understand the use of medical assistance in dying in the Québec context.

The Bronfenbrenner ecological systems theory [27] serves as the theoretical framework for this protocol (Figure 1). This theory aims to better understand a phenomenon (namely, the use of MAiD) by situating the individual in relation to the various proximal and distal environments that exert a direct or indirect influence on them. This will enable us to take into account all the specific objectives related to the 5 themes (Textbox 1), each being linked to one or more systems. Figure 1 illustrates how the Bronfenbrenner ecological systems theory will be applied to better understand the use of MAiD in the Québec context.

First, the Ontosystem refers to a person’s internal characteristics, states, strengths, and vulnerabilities. A better understanding of what characterizes people who use MAiD will enable us to address the objectives of Theme 1 relating to personal characteristics (eg, health status, social determinants of health, vulnerability factors, personal values, conception of quality of life, and relationship to suffering).

Second, the Microsystem is characterized by the person’s immediate environment, and includes the individuals with whom the person has a direct relationship (eg, relatives and health care professionals), as well as their immediate living environment, including the care setting (private dwelling, nursing home, hospital, or palliative care home). The contribution of this system to the explanation of the phenomenon will be addressed by exploring, for example, the influence of social determinants of health (eg, social support; Theme 1) and the relationship between patients and health care professionals on MAiD requests or administration (Theme 3).

Third, the Exosystem encompasses all the environments that have an indirect influence on the person. This will enable us to better understand the disparities in the use of MAiD, while considering factors associated with the organization of care and services, such as characteristics of the end-of-life care offer, depending on facilities and regions (Theme 2).

Fourth, the Macrosystem corresponds to society’s cultural matrix (eg, beliefs, values, norms, and ideologies) and includes the laws, rules, and policies that govern a society. A better understanding of the use of MAiD as a function of the Macrosystem will be addressed through several themes’ objectives, such as Theme 3 (eg, Québec societal and legal factors, social acceptability, and evolution of roles in decision-making), Theme 4 (eg, comparative analysis of laws and public policies and factors associated with the evolution of the use of MAiD in different jurisdictions), and Theme 5 (international experiences with advance requests for MAiD [AR-MAiD]).

Fifth, the Chronosystem allows for considering the temporal elements that mark the evolution of MAiD use, depending on different factors, such as the changes in the course of the disease, the changes in institutional policies, the date of coming into force of MAiD legislation, and relevant amendments.

Finally, the dotted line running through the systems represents the bidirectional influence—upward or downward—between society, its systems, and its individuals, thus demonstrating their interdependence.

## Methods

### Study Design

The diversity of research objectives calls for the use of a multimethods and convergent mixed methods design. Two complementary approaches were adopted to address the research objectives: (1) an international cross-thematical approach comprising 4 research methods and (2) 11 theme-specific methods devoted to each theme. An overview of the research methods developed for this study (n=15) is provided in Textbox 2. The different methods will be deployed in such a way that the preliminary results will inform the development of data collection tools for subsequent theme-specific methods.

**Overview of the methods according to each theme (n=15).**

**All themes**

- Cross-thematical methods
- Jurisdictions: Canada, Belgium, Switzerland, the Netherlands, the United States (California), and Australia (Victoria).
- Scoping review
- Population-based survey
- Key informant interviews
- Focus groups with health care professionals

**Personal characteristics**

- Theme-specific methods
- Individual interviews
- Case studies of triads
- Retrospective qualitative chart review

**Organization and delivery of health care in Québec**

- Theme-specific methods
- Document analysis
- System mapping

**Societal dimensions**

- Theme-specific methods
- Media coverage analysis
- Community forums

**Practices, legislation, and public policies**

- Theme-specific methods
- Comparative legal analysis on assisted dying
- Realist synthesis

**Regulation of advance requests for medical assistance in dying (MAiD)**

- Theme-specific methods
- Comparative legal analysis of advance requests for MAiD
- Mixed-literature systematic review

### Cross-Thematical Methods

#### Scoping Review

The scoping review will be conducted following the Joanna Briggs Institute methodology for scoping reviews and reported in accordance with the PRISMA-ScR (Preferred Reporting Items for Systematic Reviews and Meta-Analyses extension for Scoping Reviews) guidelines [28,29]. The scoping review will follow these steps: (1) translating the overarching objective of the study, which is to better understand the use of MAiD in Québec, into an operationalized research question, formulated to make the population, concept, and context explicit; (2) specifying inclusion and exclusion criteria; (3) in collaboration with a librarian, developing the strategy to identify relevant literature, with adaptation for 10 databases (eg, MEDLINE, CINAHL, Embase, PsycINFO, Web of Science, and gray literature); (4) conducting the selection and extraction through Covidence (Veritas Health Innovation) [30] by 2 independent reviewers; (5) presenting the findings in a narrative format, and mapping them in tables and graphs to address the review objectives; and (6) drafting recommendations and implications for research and practice in a language adapted to different target audiences (general population, decision-makers, and practitioners), and identifying future research needs.

#### Population-Based Survey

##### Study Design

A cross-sectional population-based survey on the social acceptability of MAiD in Québec, 2 other Canadian provinces, and 5 international jurisdictions (the Netherlands, Belgium, Switzerland, California [US], and Victoria [Australia]) will be conducted. In Canada, British Columbia and Ontario were selected for their demographic significance, as together with Québec, these provinces accounted for 75% of the national population in 2024 [31]. In British Columbia, the MAiD death rate ranked second after Québec, while Ontario’s rate was below the national average [6]. For their part, international jurisdictions were selected for having (1) at least 5 years of assisted dying legal permissibility (as of 2024) since a sufficiently long period is required to analyze potential explanatory factors in its growing use, and (2) different forms of assisted dying (euthanasia and physician-assisted suicide) for comparability purposes. The survey will be conducted among the adult population (18 years of age or older), in the official languages of the selected jurisdictions (either French, English, Dutch, German, or Italian). The target sample size is calculated according to the following parameters: a 95% CI, a maximum proportion of 0.50, a margin of error of ±5%, multiplied by the number of jurisdictions (n=8). A larger sample of respondents will be selected for Québec to allow regional comparisons (expected total n=3580).

##### Data Collection

The questionnaire, based on and adapted from questionnaires designed by CIRAMM members [25,32-34], will include acceptability and knowledge of MAiD, palliative care, and continuous palliative sedation, previous experiences with MAiD and with other end-of-life care, social determinants of health, and sociodemographic characteristics. Special consideration will be given to tailoring the level of language and MAiD-related terminology to the specific context of each jurisdiction, while preserving thematic consistency across all settings. The survey will be conducted online via a web panel designed by the chosen polling firm (to be determined).

##### Data Analysis

Data will be weighted according to sociodemographic characteristics to adjust results to represent the target population. Usual descriptive statistics will be used to summarize the factors associated with the acceptability of MAiD and with the use of MAiD for the selected jurisdictions. Multivariable regressions will be conducted to identify which factors (knowledge, previous experiences, social determinants of health, or sociodemographic characteristics) are associated with the acceptability of MAiD and contribute to the differences in use of MAiD across jurisdictions.

#### Key Informant Interviews

##### Study Design

To complement the results of the scoping review with data specific to Québec and each abovementioned jurisdictions, a qualitative descriptive study will be carried out on the factors associated with the growing use of MAiD. A total of 50 key informants will be selected for their expertise and experience in the field of MAiD in the abovementioned jurisdictions. We will include all MAiD program managers in Québec, a population of approximately 30 individuals, in order to gain a comprehensive understanding of the situation across the province. In addition, we will include members of MAiD regulatory authorities (eg, members of an oversight body or professional college) or people actively involved in MAiD practice (eg, MAiD program manager or experienced provider). Selection will be done by a nonprobabilistic maximum variation purposive sampling method to obtain rich and meaningful information, as well as to capture the breadth of different experiences and perspectives [35].

##### Data Collection

We will conduct semistructured interviews with participants in order to gain insight into potential explanatory factors for the use of MAiD. The interview guide, available in French and English, will be pretested and adapted in an evolving and iterative process throughout the data collection process [36]. The interview guide is available in Multimedia Appendix 1. Recruitment in Québec will take place through networks already established by CIRAMM members (ie, communities of practice of Québec MAiD providers and support structures for MAiD, among others) [37-42]. In other provinces of Canada and internationally, we will use a snowball approach [43] and mobilize our many Canadian and international networks: medical associations and federations, professional regulatory bodies, etc [44-47]. Interviews will be conducted by trained interviewers via the online Microsoft Teams platform. They will be recorded, and research notes will be taken by the interviewers to synthesize important ideas.

##### Data Analysis

The transcription process will leverage Teams’ automatic transcription system, followed by editing by the interviewers. A descriptive thematic analysis will be conducted on the transcripts and research notes [48,49], with an iterative review process to refine codes based on the interview guide. Coding disagreements will be discussed between coders until a consensus is reached. The QDA Miner software (Provalis Research) [50] will support the coding process, allowing for the identification of recurring themes across participants’ discourse.

Our study is guided by the quality criteria articulated in Tracy’s [51] qualitative research framework. Specifically, our approach is informed by the 8 key markers: worthy topic, rich rigor, sincerity, credibility, resonance, significant contribution, ethics, and meaningful coherence, while allowing flexibility, reflexivity, and interpretive dialogue throughout the research process (as for all the qualitative analyses included in this protocol).

#### Focus Groups With Professionals Involved in MAiD

##### Study Design

We will explore, with focus groups, the viewpoints of professionals involved in MAiD in the same jurisdictions as the population-based survey and interviews with key informants.

##### Participants

Physicians and nurse practitioners involved in assessing, providing, or supporting families accessing MAiD will be recruited (nurses, social workers, spiritual care workers, ethicists, etc). Professionals must have participated in at least 10 MAiD cases or have been involved in MAiD for at least 2 years. We will recruit the professionals using a nonprobabilistic purposive sampling to obtain maximum variation (gender, age, experience, location, and type of practice, etc) [35].

##### Data Collection

A total of 18 online focus groups, each comprising of 6-8 participants, and lasting about 2 hours, will be organized as follows: 4 groups in Québec (physicians and nurse practitioners: n=2; other professionals: n=2); 4 groups in the rest of Canada (physicians and nurse practitioners: n=2; other professionals: n=2); 2 groups in each international jurisdiction (Netherlands: n=2, Belgium: n=2, Switzerland: n=2, California: n=2, and Victoria: n=2). The international focus groups will consist of only physicians because of the limited involvement of other health care professionals in MAiD. We will construct the facilitation guide (with the help of the international advisory committee for jurisdictions other than Québec) based on information extracted from the key informant interviews. The facilitation guide will be pretested with a group of 4 people familiar with the reality of MAiD, but not approached as potential participants (Multimedia Appendix 2). A preliminary version of the facilitation guide can be found in Multimedia Appendix 1. For recruitment, we will use the same methods as for the key informant interviews. Focus groups will be conducted virtually, recorded, and transcribed via Microsoft Teams.

##### Data Analysis

Analysis of the qualitative data from the focus groups will follow the same procedure as for the key informant interviews.

### Theme-Specific Methods

#### Overview

In this section, we will present the 5 themes that structure the call for proposals and their associated specific methods. For the sake of consistency, we have chosen to present them in accordance with the Bronfenbrenner ecological systems theory, from the microsystem (eg, personal characteristics) to the macrosystem (eg, practices, legislation, and public policies) as presented in Figure 1.

#### Theme 1: Personal Characteristics

This theme focuses on the personal characteristics of people who have requested MAiD, using qualitative methods to help understand the process that led to their request.

##### Study Design

The following three complementary methods will be used: (1) individual interviews with people requesting MAiD; (2) case studies of triads involving interviews with people requesting MAiD, one of their close-ones, and a member of their caregiving staff; and (3) a retrospective chart review.

##### Individual Interviews

#### Participants

We will conduct interviews inspired by narrative approaches of the “life story” [52-55] with approximately 30 people who have requested MAiD, diversifying the choice of participants according to their personal characteristics (age, gender, identity, cultural or ethnic origin, diagnoses, disability status, and socioeconomic situation).

#### Data Collection

A semidirective approach will be used to focus on the stories collected, while directing them toward the central themes, encouraging the emergence of new reflections. One or two interviews will be conducted with each participant (varying in length from 30 to 60 minutes, depending on the participant’s health and ability to participate). The interviews will be recorded and transcribed integrally. Interviewers will keep a diary in order to note their impressions following each interview and use this information to enrich their analysis (emotional state of the participant, nonverbal cues during the interview, general context of the interview, etc). Recruitment will be based on the CIRAMM’s contacts across the province (MAiD interdisciplinary support groups [ISGs], hospice care organizations, patient associations, etc) with the aim of reaching participants in different regions of Québec (urban, semiurban, and rural). During recruitment, we will explore the possibility of involving one of the participant’s close-ones, as well as one of their care providers, in order to conduct a triadic case study (see “Case Studies of Triads” section below).

#### Data Analysis

A thematic analysis of the data [56,57] will be conducted according to the following steps: (1) interview verbatims and diaries will be read, (2) a sample of 3 transcripts will be randomly chosen and analyzed to produce a list of themes, and (3) the content of the remaining interviews and diaries will be classified from this list of themes using NVivo software (version 14; Lumivero; released in 2023). The quality of the codification process will be ensured by an interjudge agreement process in accordance with validity and quality criteria in qualitative research [58].

### Case Studies of Triads

#### Overview

Many studies [59,60] focus on the individual perspectives of people who want to receive assistance in dying, their relatives, or caregiving staff. This approach does not reveal the relational dynamics at play within a request, the contrasts between different individual perspectives, and the way these factors influence each individual’s perspective.

#### Participants and Data Collection

To overcome the shortcomings identified in current literature, we will carry out 10 triadic case studies, each comprising “contextualized narrative” interviews with the person requesting MAiD, one of their close-ones, and one of their care providers (doctor, nurse, allied health clinicians, care assistant, etc). This method was tested in another study conducted in the Netherlands by a coleader of this theme and her colleagues [61]. A different interview guide will be used for each subgroup, aimed at gaining a better understanding of the personal factors that motivate people to request MAiD from the perspective of the 3 subgroups. Each person will be interviewed individually.

#### Data Analysis

An analysis anchored in phenomenology, combining a longitudinal and multiperspective framework [62], as well as different aspects of reflective lifeworld research [63], will be used [62,64,65]. The whole process will be iterative and guided by multiple discussions between the members of our research team [66].

### Retrospective Qualitative Chart Review

#### Overview

The researchers will also undertake a retrospective chart review of MAiD requesters in a single institution in Montréal. The purpose of this study is to better understand the role of economic disadvantage (which is defined as dependence on social assistance as one’s sole form of revenue) on these individuals’ MAiD requests, particularly for those requesters whose natural death is not reasonably foreseeable (Track 2), as it is this group where public debate about the impact of economic disadvantage on requests has arisen. This part of the protocol draws upon similar methods used in 2 studies currently being carried out by CIRAMM researchers, the first involving a population of requesters in which the assessment required a psychiatric consultation [67], the second involving people with amyotrophic lateral sclerosis [68].

#### Data Collection and Analysis

We aim to review the charts of all Track 2 requesters from 2021 to 2025 who are experiencing economic disadvantage. We will obtain a list of all requesters at the identified institution. We will then do a preliminary review to determine the economic situation of each requester, identifying those in a situation of economic disadvantage. The charts of this subgroup will be subject to a complete chart review. First, we will extract data relating to each individual’s sociodemographic profile: age, gender, social circumstance, diagnoses, stated reason for requesting MAiD, and whether MAiD was ultimately received. Second, we will review all notes pertaining to the person’s request for an assisted death. We will extract all segments that refer to the person’s statements about assisted dying, as well as the writer’s views about those statements, if any. We will be particularly attentive to any notes relating to the person’s economic situation and its potential impact on the experience of their disease, access to care, and suffering. In our analysis, we will focus on the manifest content of the extracted text. We will identify meaning units from the extracted segments, grouping them together in categories. Ultimately, we will seek patterns that link the categories, which describe and explain the role of a person’s economic circumstances in their request for an assisted death, with their sociodemographic profiles, recognizing that a plurality of such narratives may emerge from these records [69].

### Theme 2: Health Care Organization and Delivery in Québec

#### Overview

For this theme, we adopt a systematic comparison approach between Québec regions to examine the relationships between gaps in the use of MAiD and differences in sociodemographic profiles, in health services structures, practices, and perceptions. Québec regions will be further broken into territories corresponding to each of the 30 MAiD ISGs.

#### Study Design

A mixed methods approach will be implemented, including a document analysis and system mapping. Qualitative discourse analysis will be used to summarize government reports on MAiD (provincial and regional) and information documents available to the public, to explain how MAiD is presented and how resources are promoted in each region. Correlational analysis from MAiD reports and regional sociodemographics will be used to explore the strength and direction of links between regional MAiD rates and the social profile of the population. Both qualitative and quantitative results will be mobilized for a system mapping analysis to show possible causal loops between different categories of items.

#### Document Analysis

Document analysis is a systematic procedure for reviewing and synthesizing various sources (printed or digital), including their format dimensions (institutional authors, aims, and availability), and their discourse (themes, perspective, posture, and legitimation).

#### Data Collection

We will first create a mixed database of organizational documents, records, and reports produced by the ISGs on cases of refused, accepted, abandoned, and administered MAiD requests for the period 2019-2025 (case profiles including age of individuals, status of request, trajectory characteristics, access to alternative curative, palliative, and psychosocial care and services). Data gathered by the Québec End-of-Life Commission (Control Commission) will be considered, as well as sociodemographic data by region (population, age, socioeconomic profile, disease incidence, deaths, and causes of death, including rates of unassisted suicide). Other documents from gray literature will also be analyzed (local reports and registers of care and services from the ISGs, the End-of-Life Commission, and the Ministry of Health and Social Services, information and training documents, etc).

#### Data Analysis

In order to produce a general portrait of the evolution and current state of structures, resources, and practices in each region, the qualitative data will be subject to a documentary analysis using the read, extract, analyze, and distill method [70]. Quantitative data will be the subject of descriptive and comparative statistical analyses of regional realities and their evolution (averages, frequencies, and SDs), population representativeness of cases, as well as correlation analyses between the explanatory variables and the rate of increase in MAiD use. The analysis of the proportionality of the variables for each region will be used, among other things, to determine the associations and the relative weight of population aging in the use of MAiD, which is the main assumption made in the CSFV report [4].

#### System Mapping

##### Data Collection

Qualitative and quantitative analyses will be synthesized into a system mapping model, an approach for integrating systems analysis into the implementation of public and community health programs, care, and services using visual representations [71,72].

##### Data Analysis

This method has the advantage of facilitating the detailed description of complex systems by interested parties and producing a rigorous evaluation of the concrete impacts of an intervention, policy, or program and their local variations [73]. System mapping allows the description and comparison of systems and organization models to produce conceptual maps, diagrams, and causal loops reflecting complex decision-making and accessibility processes [74]. All these analyses will enable us to meet our objectives by modeling the organization of care and services for each region, creating detailed statistical reports as well as typologies of practice models and individual pathways [75].

### Theme 3: Societal Aspects

#### Study Design

This theme encompasses 2 methods. First, media coverage analysis can provide access to the discursive and symbolic predominance of MAiD and, in doing so, shed light on how content contributes to ways of thinking, feeling, and acting about MAiD, and how we integrate it into our “own social value systems” [76]. Second, community forums (CFs) favor an open, participatory approach with citizens and take the pulse of the community’s concerns, knowledge, and resources [77,78]. In addition to stimulating the exchange of ideas, this dynamic method brings together people from various backgrounds in a given region to hear and gather their opinions on a subject that concerns them, such as the use of MAiD.

#### Media Coverage Analysis

##### Data Collection

The main French- and English-language media published in each of the studied regions of Québec will be analyzed (Journal de Québec, Journal de Montréal, The Gazette, La Presse, Le Devoir, Hebdo Rive-Nord, Journal de Joliette, le Laurentien, le Soleil, and Le Nouvelliste et le Nord-Côtier) from 2014 (the year the Act respecting end-of-life care was adopted in Québec) to the present. A systematic search on Eureka will be carried out with the help of a librarian, by combining corollary terms referring to MAiD. All journalistic articles on MAiD will be included. Two people will select the texts and extract the data using Covidence [30].

##### Data Analysis

The qualitative discourse will be triangulated through an inductive thematic analysis using NVivo 14 software [79], and a lexicographic analysis [80] conducted with IRaMuTeQ (Pierre Ratinaud) [81]. Media texts, in the form of a textual corpus, will be statistically contrasted in the light of meaningful lexical categories derived from the software’s statistical calculations (ie, correspondence factor analysis and top-down hierarchical classification method). This quantitative input will validate and/or complement the discourse analysis initially carried out.

#### Community Forums

##### Participants

Adult citizens of a given region will be invited to participate in CFs. The team will use a variety of means to encourage diversity in the participants’ sociodemographic profile (gender, sex, age, sexual orientation, ethnic group, socioeconomic status, level of education, religious practices, beliefs, spirituality, and ability and disability). For each CF, we set no maximum number of participants, but anticipate a minimum of 20 representatives per region. The 6 CFs will be organized virtually or in person in the 3 regions with the highest annual proportion of MAiD deaths (Lanaudière, Bas-Saint-Laurent, and Capitale-Nationale), and the 3 regions with the lowest proportion of MAiD deaths (Montréal, Mauricie-Centre-du-Québec, and Côte-Nord) [4]. These regions also represent urban, semiurban, and rural environments. In order to represent and understand the Indigenous perspective on palliative care and MAiD, we plan to consult Nunavik citizens, in coconstruction with community representatives.

##### Data Collection

The facilitation guide will be elaborated on the basis of the results of the cross-thematical methods and in coconstruction with our partner organizations, patients, and relatives. Recruitment will be based on a call for applications via social media, health care associations, distribution lists of community, associative, and organizational partners, etc. Individuals will be able to register using an online form. Questions about their sociodemographic profile will enable us to assess the number and diversity of people attending, and to adjust accordingly. CFs will be conducted online via Microsoft Teams or face-to-face, and moderated by the researchers. Discussions will be recorded and automatically transcribed by Microsoft Teams, with subsequent verification. Observation notes will be taken by research assistants, and a logbook kept by each of the facilitating researchers will help contextualize the data.

##### Data Analysis

The data collected will be analyzed using an inductive thematic approach for each region. This qualitative analysis will then be compared with a quantitative content analysis under the same methodology as the media coverage analysis (ie, triangulated through IRaMuTeQ with a lexicographic analysis of the discussion transcriptions).

### Theme 4: Comparison of Practices, Legislation, and Public Policies

#### Comparative Legal Analysis on Assisted Dying

Legal databases (eg, Société québécoise d'information juridique, Centre d'accès à l'information juridique, and La Référence for Québec; Canadian Legal Information Institute for the rest of Canada; and Lexis, Westlaw, and HeinOnline for selected international jurisdictions) will be searched to identify legal documents associated with MAiD legislation for the jurisdictions selected. The comparative positivist legal analysis (legislation, case law, and doctrine) carried out by our team of legal experts will enable us to identify the differences and similarities between laws and the scope of legislative criteria, and to grasp the appropriateness of their interpretation by contrasting them with the findings emerging from the scoping review. This work will be complemented by a comparative analysis of Québec public policies promoting the quality of life of sick people and their families (eg, home care [82] and informal care [83]), as well as of the various legal mechanisms contained in the Québec legislation (MAiD, advance directives, palliative care, and continuous palliative sedation), while considering recent updates to the law. In addition, by compiling (and critically comparing) data from 2 complementary sources [84], we will be able to address “Examine how MAiD numbers in Québec compare to those in other jurisdictions” objective, which is to examine how MAiD numbers in Québec compare to those in other jurisdictions: (1) the annual reports of the Control Commissions of the selected jurisdictions based on official MAiD declarations, and (2) independent surveys conducted in certain jurisdictions and aimed at documenting the frequency of MAiD practices, in addition to other types of end-of-life medical practices [85,86].

#### Realist Synthesis

The final objective of Theme 4 (Textbox 1 [Explore how Québec’s context can explain differences in MAiD use]) represents one of the overarching goals of the project, requiring the consideration of all results generated across themes, through both cross-thematic and theme-specific methods. To this end, we will draw on the realist synthesis method developed by Pawson et al [87]. The realist synthesis method addresses questions concerning what works for whom, in what circumstances, in what respects, and how a complex social intervention operates, such as a public policy, a health care system, or even a law [87-89].

Realistic synthesis is characterized by (1) the use of a conceptual framework and (2) the involvement of an advisory committee at various stages of the process. The conceptual framework chosen will combine the Bronfenbrenner ecological systems theory and that of the Health System Performance Measurement [90]. It will be adapted to the phenomenon under study (use of MAiD) and will provide a global understanding of the explanatory factors and the underlying mechanisms. In particular, the framework will consider (1) the characteristics and inputs of the legislation (macrosystem) and its operational functioning (2) the characteristics and social determinants of health related to individuals (microsystem) who use MAiD, (3) the outputs of the system (exosystem), which include access to and provision of MAiD and associated services, and (4) the results obtained (the use of MAiD in terms of requests and provisions). We will add the historical context (chronosystem) as a potential explanatory factor for the differences observed in the evolution of the use of MAiD (eg, legislative change initiated by whom and how).

A bipartite advisory committee will be set up, made up of (1) members of an international advisory committee and (2) representatives of the stakeholders (patients and their families, physicians, nurse practitioners, and other health care professionals, managers, and decision-makers) involved in MAiD in Québec. The involvement of the bipartite advisory committee will be particularly decisive in adapting the conceptual framework to the specific characteristics of the MAiD study, as well as in interpreting the results. The comparison of our data, based on the conceptual framework, will enable us to identify the contributing factors for which data already exist, as well as those for which further studies are required.

### Theme 5: Regulation of AR-MAiD

#### Overview

On June 7, 2023, Québec’s Act respecting end-of-life care was modified to allow AR-MAiD from people diagnosed with a disease leading to decisional incapacity [2]. Legal dispositions related to AR-MAiD came into effect on October 30, 2024. AR-MAiD may lead to further increases in the use of MAiD, given its widespread acceptability in Québec [26,33,91]. On the other hand, providing MAiD to patients who are no longer competent, based on their advance requests, can be challenging to implement. To inform the implementation of the AR-MAiD regime in Québec, a comparative analysis of the legal regimes of jurisdictions that already allow such requests and a systematic review of implementation challenges will be conducted.

#### Comparative Legal Analysis on AR-MAiD

A comparative portrait specific to AR-MAiD will be drawn from the legal analysis conducted under Theme 4. Results will highlight processes that led to AR-MAiD legalization in permissive jurisdictions and reasons underlying different legislative choices pertaining to eligibility criteria, procedural safeguards, and the assessment of suffering. Professional standards established by medical associations, learned societies, and regulatory bodies will complement our legal interpretation work [92]. Legal analysis specific to this theme will be anchored in a recent review of Dutch and Belgian AR-MAiD legislation [92], which we will extend to include Luxembourg, Colombia, Spain, and Québec.

#### Mixed-Literature Systematic Review

We aim to provide a comprehensive overview of international literature on the barriers and facilitators (both anticipated and experienced) related to AR-MAiD. Perspectives from patients, their relatives, health care professionals, legal experts, and scholars will be sought. Primary studies (qualitative, quantitative, and mixed methods studies), theoretical work, as well as relevant documents from gray literature (eg, theses and Control Commissions’ reports) will be considered for inclusion. Peer-reviewed studies published after 2002 will be searched by a librarian in 10 electronic databases, with no restriction on language or origin of the authors. Screening of retrieved citations, assessment of the methodological quality of included studies, and data extraction will be conducted independently by pairs of researchers, supported by the software Covidence [30]. A preliminary search for publications on AR-MAiD revealed a predominance of qualitative studies, with a few surveys and mixed methods studies [93-95]. Hence, to enable synthesis and integration, quantitative data will be transformed into themes and subthemes in a convergent integrated approach. Identified barriers and facilitators to implementing AR-MAiD will be summarized following a content analysis of included documents, examined in light of the Québec regime, and contrasted according to (1) whether they are anticipated or truly experienced, (2) the perspective from which they originate (eg, that of health care professionals or relatives), (3) when they might arise along the MAiD process (request drafting, eligibility assessment, or MAiD provision), and (4) prevailing legislation and codes of practice. We expect our legal analysis and systematic review to highlight significant challenges to implementing AR-MAiD in Québec and also possible ways to overcome them.

### Governance and Methods Sequence

**Figure 2:**
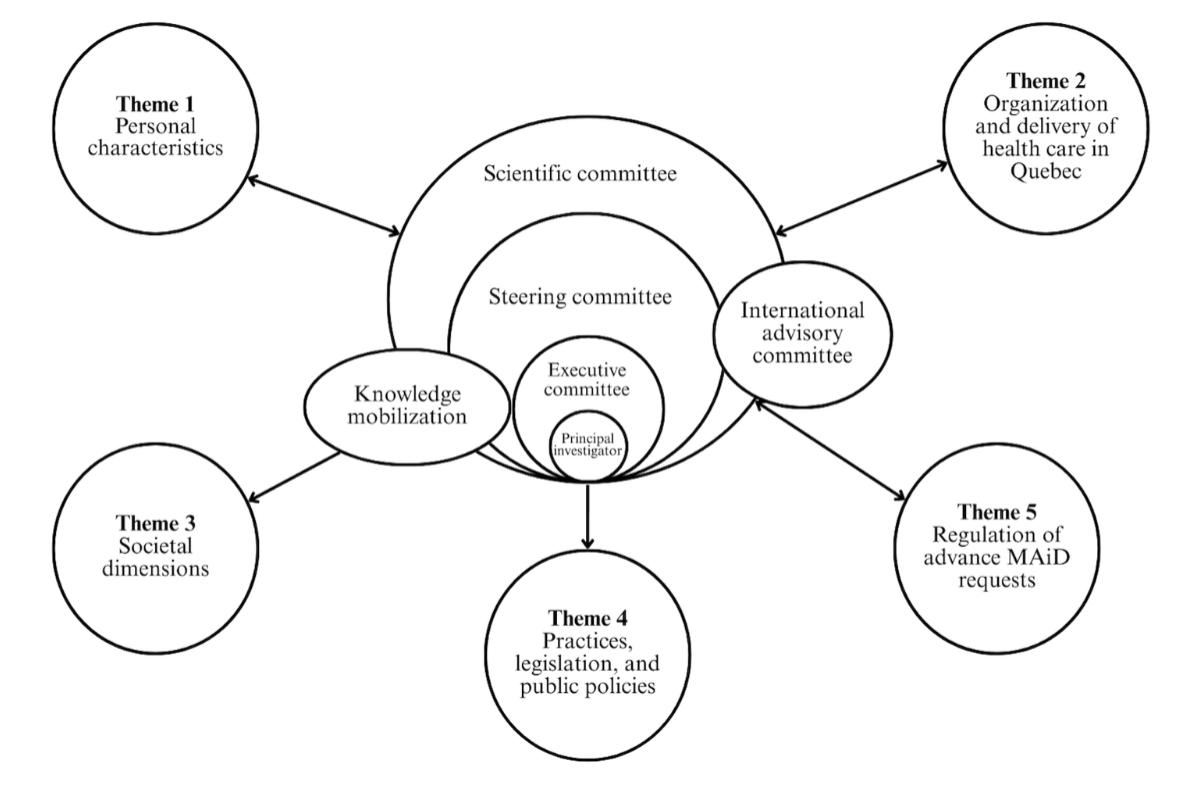
Consortium interdisciplinaire de recherche sur l'aide médicale à mourir governance structure. MAiD: medical assistance in dying.

To meet the challenge of this mixed methods and multimethods study, CIRAMM rallied leading forces in the field of end-of-life care: 46 coresearchers (Québec, Canada, and international), 14 clinicians, 5 partner organizations, and 10 postdoctoral fellows and graduate students. Researchers are drawn from over 15 disciplines and work in various university departments, programs, and faculties: medicine (palliative care, family medicine, geriatrics, neurology, psychiatry, emergency medicine, and pediatrics), nursing, law, psychology, clinical ethics, epidemiology, social work, occupational therapy, pharmacy, philosophy, anthropology, religious sciences, and theology, sociology, and population health. We developed a governance structure to support CIRAMM members, enabling us to successfully coordinate the various research subteams (Figure 2). We set up an executive committee responsible for the operationalization and general coordination of CIRAMM. A steering committee made up of clinicians, researchers, and patient partners supports the executive committee in general decisions and orientations. A scientific committee brings together all researchers in charge of the specific themes, whose subteams will meet as projects progress. We also set up an international advisory committee made up of 10 collaborators (Canadian and international researchers) with expertise in euthanasia, assisted suicide, and end-of-life care.

Each theme has a subteam responsible for its theme-specific methods. Coordination tools for the deployment sequence of methods are put in place to structure and adapt the activities of the study as it evolves.

### Knowledge Mobilization

Inspired by the Knowledge-to-Action framework of Graham et al [96], we aim not only to produce appropriate knowledge adapted to the target audiences, but also to produce this knowledge by, for, and with them. The strategy put forward for this study is intended to be inclusive and collaborative at all stages of the research. To carry out this mission, we put together a knowledge mobilization committee, including patients, their close-ones, and key stakeholders, to play an anchoring role and to (1) support the subteam in the production of knowledge transfer (KT) tools, (2) plan the project’s mobilization and KT activities with partners and users, (3) coordinate deliverables with preidentified milestones, (4) coordinate a unified plan for disseminating and promoting KT activities among partners, and (5) provide feedback on the activities developed, assessing whether the strategy’s objectives have been met, while making adjustments where necessary.

### Ethical Considerations

In Canada, ethical review by a research ethics board is a mandatory requirement prior to the commencement of any research involving human participants or human biological materials [97]. The ethics approval of the study will be done in stages, given the planned sequence of the various methods. So far, we have received ethical approval from the Scientific and Research Ethics Committee of the Centre intégré de santé et de services sociaux de Laval (MP-35-2025-1112) and the Research Ethics Committee on Health Science at the University of Montréal (MP-35-2025-1112) for the cross-sectional methods of interviews with key informants and focus groups with professionals. As each methodological component involves distinct procedures, details regarding informed consent, data protection and confidentiality measures, and participant compensation will be described in the corresponding articles reporting on each method.

## Results

The study was funded for 3 years and started in July 2024. We initiated projects that do not require the approval of an ethics committee because they do not involve human participants: scoping review, realist synthesis, both comparative legal analyses, mixed-literature systematic review, media coverage analysis, and document analysis. Detailed protocols for the scoping review and the mixed-literature systematic review have been drafted and submitted for publication.

The only method for which we have results to report to date is the interviews with key informants. We completed the interviews in August 2025 and the qualitative analysis in September 2025. The study sample included 63 participants, representing a diverse yet Québec-centered group of stakeholders involved in MAiD. Most participants were based in Québec (n=48), reflecting the primary focus of the study, while a smaller number came from other Canadian provinces (n=5) and international jurisdictions where assisted dying is permitted, including Belgium, the Netherlands, Switzerland, Australia, and the United States (n=2 each). In terms of roles related to MAiD, participants primarily included individuals responsible for MAiD support or coordination structures (n=38), followed by MAiD providers (n=15), experts in the field (n=14), and representatives of regulatory bodies (n=7). This composition ensured the inclusion of complementary perspectives spanning clinical practice, organizational coordination, expertise, and regulatory oversight. We are currently drafting a manuscript reporting the results for publication.

The focus groups are currently underway (December 2025). Ethical approval was obtained in August 2025. All the other subteams are preparing for ethics approval (protocol, data collection tools, consent forms, etc). This includes individual interviews, case studies of triads, retrospective qualitative chart review, CFs, and population-based survey methods.

## Discussion

### A Better Understanding of the Use of MAiD

This study is the first to comprehensively examine the use of MAiD in Québec. It builds on existing evidence and attempts to fill knowledge gaps. Empirical work aimed at explaining the use of MAiD remains uneven and conceptually dispersed. The literature tends to address isolated aspects of MAiD, often yielding divergent findings and rarely bridging legal frameworks, organizational contexts, societal dynamics, and patient-level factors. A more comprehensive and context-sensitive analysis is therefore required, particularly within the Canadian and Québec settings.

The results will provide a global understanding of the factors explaining the evolution of the use of MAiD, both by cross-thematical methods and by theme-specific methods. Our choice of the Bronfenbrenner ecological systems theory [27] will provide a better understanding of the use of MAiD by situating the individual in relation to the various proximal and distal environments that exert a direct or indirect influence on them.

Accordingly, our multiple methods will allow us to document each level—from the individual to the collective—while accounting for internal and external factors as well as the temporal dimension. We will be able to compare these data with those from other jurisdictions and to triangulate them in order to obtain a richer perspective on this phenomenon.

While focusing on explaining the high uptake of MAiD in Québec, findings could also provide insight into reasons for a decline in demand (if any) or lower rates of deaths by MAiD observed in other jurisdictions [84]. Several hypotheses exist to explain the increase in the use of MAiD, but much still needs to be done to understand this choice made by thousands of people every year.

### Strengths and Limitations

The strengths of this study include (1) the use of a variety of methodologies, allowing for methodological triangulation that promotes reliability of the results; (2) the breadth of this interdisciplinary team with its combined set of skills and an international scientific committee ensuring the feasibility of the project; (3) the creation of the knowledge mobilization committee disseminating the results to various target audiences; and (4) the diversity of activities proposed in the study to train the next generation of students in end-of-life research.

Among the limitations, 2 of the selected jurisdictions (Belgium and Switzerland) are multilingual (other than French and English), and, within their respective linguistic communities, the rates of assisted suicide or euthanasia are quite different. It will be very challenging to compare the data, given the linguistic and cultural differences. Also, the evolution of MAiD rates is perceptible in some countries, while not in others. Alternative explanations would need to be taken into consideration. For example, the number of cases reported to the authorities may differ from the actual number of cases. It will also be necessary to consider the evolution of alternatives to MAiD, particularly continuous palliative sedation, which is experiencing an important growth in some countries, notably in the Netherlands and Switzerland [98]. Finally, large and comparative population surveys in different jurisdictions present several scientific and practical challenges that will require rigor and attention to detail from our research team.

## Appendix

Multimedia Appendix 1 Interview guide, key informants.

Multimedia Appendix 2 Discussion group facilitation guide.

Multimedia Appendix 3 Peer review report from Fonds de recherche du Québec - Société et culture (FRQSC).

## Abbreviations

AR-MAiD: advance requests for medical assistance in dying
CF: community forum
CIRAMM: Consortium interdisciplinaire de recherche sur l'aide médicale à mourir
ISG: interdisciplinary support group
KT: knowledge transfer
MAiD: medical assistance in dying
PRISMA-ScR: Preferred Reporting Items for Systematic Reviews and Meta-Analyses extension for Scoping Reviews

## Data Availability

Some anonymized data may be shared upon request, within the limits authorized by the research ethics committee.
