# Supplementary material for: Understanding the Factors Explaining the Growing Use of Medical Assistance in Dying in Québec: Protocol for an Interdisciplinary Mixed Methods and Multimethods Study
Source: JMIR Res Protoc. 2026 Apr 20;15:e83549. doi: 10.2196/83549 (PMC13139836; doi:10.2196/83549)
Supplement: Multimedia Appendix 1 [file resprot_v15i1e83549_app1.pdf]

This is a Multimedia Appendix to a full manuscript published in the J Med Internet Res.
For full copyright and citation information see http://dx.doi.org/10.2196/jmir.83549

Interview guide – Key informant		Quebec			Canada			International
		GIS	Providers	Organization	Providers	Healthcare managers	Organisation	International
General Hypotheses About the Use of MAiD (this term includes euthanasia and assisted suicide) in Québec<br>Based on your experience, what could explain the high increase in the use of MAiD in Quebec compared to elsewhere?		x	x	x				
Specific themes								
Practices, Laws, and Public Policies	Over the past years, have you observed an increase or decrease in MAiD requests and provisions in your setting/region/jurisdiction?<br>What factors do you think could explain this trend?				x	x	x	x
Practices, Laws, and Public Policies	Would you say that MAiD is integrated into end-of-life care in your setting? If so, how?	x	x	x	x	x	x	x
Practices, Laws, and Public Policies	In your setting, do you encounter challenges related to the interpretation or assessment of MAiD eligibility criteria? If so, could you specify these challenges?<br>*e.g., illness/disability, decline, suffering, capacity to consent, free and informed consent, GRD or NGRD (natural or non-natural foreseeable death), etc*	x	x	x	x	x	x	x
Societal Level	*If any, what debates/tensions exist in your setting/region/jurisdiction regarding MAiD legislation?*<br>**Ask to specify at what level their answer applies (practice setting, province, country, etc.).**	x	x	x	x	x	x	x
Organization of Care and Services	Can you describe how the MAiD process unfolds in your setting/region/jurisdiction, from the request to assessment and provision?<br>*Is there a centralized or decentralized approach to services?*	x	x		x	x		x
Organization of Care and Services	Based on your experience, how does the organization of care and services in your setting/region/jurisdiction influence the use of MAiD?	x	x		x	x		x

	Regarding MAiD support structures:<br>• In the pre-interview questionnaire, you mentioned (response to question 16).<br>• What role does this support structure play?<br>• How does this support structure influence the use of MAiD?	x	x	x	x	x	x	x
	Regarding MAiD-dedicated teams or professionals:<br>• In the pre-interview questionnaire (response to question 17):<br>• What role do these dedicated teams or professionals play?<br>• How does their practice (or their absence) influence the use of MAiD?	x	x	x	x	x	x	x
	Regarding service wait times:<br>• In the pre-interview questionnaire (question 22):<br>• Based on your experience, what influence do wait times have on the use of MAiD?	x	x	x	x	x	x	x
	Regarding undeclared MAiD, euthanasia, or assisted suicide cases:<br>• In the pre-interview questionnaire, you indicated that (response to question 19).<br>• What led you to this conclusion? What are your reasons or observations?	x	x	x	x	x	x	x
Personal Characteristics	Based on your experience, what personal characteristics are associated with a greater likelihood of requesting MAiD?<br>*(e.g., health status, social determinants of health, vulnerability factors, quality of life, relationship to suffering, etc.)*	x	x	x	x	x	x	x
Advance Requests for MAiD	How is the arrival of advance requests for MAiD perceived in your setting/region/jurisdiction (by users, families, caregivers, managers, etc.)?	x	x	x				P-B
	Based on your experience, what issues could arise along the care trajectory of a person diagnosed with an illness leading to incapacity (e.g., dementia) from the formulation of an advance request for MAiD to its assessment and provision?	x	x	x	x	x	x	P-B
	Based on your experience, what issues could arise regarding family members during the process of advance requests for MAiD?<br>Is the role of the trusted third party well-defined, and what challenges might this present in practice?	x	x	x	x	x	x	P-B
Conclusion	How do you envision the practice of MAiD evolving in the future in your setting/province/jurisdiction?	x	x	x	x	x	x	x
	Are there any elements we have not discussed that you would like to add?	x	x	x	x	x	x	x
	May we contact you if we need additional clarifications or further questions?	x	x	x	x	x	x	x
