# Supplementary material for: Understanding the Factors Explaining the Growing Use of Medical Assistance in Dying in Québec: Protocol for an Interdisciplinary Mixed Methods and Multimethods Study
Source: JMIR Res Protoc. 2026 Apr 20;15:e83549. doi: 10.2196/83549 (PMC13139836; doi:10.2196/83549)
Supplement: Multimedia Appendix 2 [file resprot_v15i1e83549_app2.pdf]

Centre intégré de santé et de services sociaux Québec

Université de Montréal

[preliminary]

This is a Multimedia Appendix to a full manuscript published in the J Med Internet Res.
For full copyright and citation information see http://dx.doi.org/10.2196/jmir.83549

Discussion Group Facilitation Guide

General Research Objective: Better understand the use of medical assistance in dying (MAiD) in the Quebec context.

Roundtable Presentation + Contextualization	
Societal Level	Based on your experience, what societal factors (values, norms) might contribute to the evolution of MAiD requests and its administration?<br>Based on your experience, would you say there is a "social acceptability" of MAiD in Quebec/in your province/jurisdiction? What factors might explain this level of social acceptability?<br>**Ask participants to specify at what level their answer applies (practice setting, province, country, etc.)*
Practice, Laws and Public Policies	Based on your experience, what elements of the law (legal status of MAiD as a form of care, interpretation of eligibility criteria, safeguards) can contribute to the evolution of MAiD requests and its administration?
Organization of Care and Services	Based on your experience, does the organization of care and services in your workplace/province/jurisdiction influence the use of MAiD? Please elaborate.<br>Based on your experience, how can the variability in the use of MAiD between institutions or regions within the same workplace/province/jurisdiction be explained?<br>Based on your experience, what does "having access to palliative care" mean? Can this access influence the use of MAiD?
Personal Characteristics	Based on your experience, what characteristics or personal experiences are associated with a greater or smaller likelihood of requesting (and receiving) MAiD? *(e.g., health status, social determinants of health, vulnerability factors, care trajectory, types of suffering experienced, perceived quality of life, having accompanied a loved one through MAiD, not having any caregivers, etc.)*<br>**Ask the same question for other end-of-life and palliative care practices.*
Advance Requests for MAiD	Based on your experience, what are the main reflections, issues and, observations related to MAiD advance requests in your workplace/province/jurisdiction?<br>Sub-question:<br>- In your opinion, is the use of MAiD advance requests likely to follow the same trend as the use of "contemporary" MAiD? Why?
Conclusion	Besides the elements discussed, are there any other factors that could explain the increased use of MAiD in Quebec compared to other jurisdictions?<br>How do you envision the practice of MAiD in the future in your workplace/province/jurisdiction?<br>Is there anything we haven't discussed that you would like to mention?

Legend

**The terminology "use of MAiD" refers to both MAiD requests and administration*

In blue: clarification for participants from Quebec
In red: clarification for participants from the rest of Canada
In green: clarification for participants from jurisdictions other than Quebec and Canada
