# Supplementary material for: Understanding the Factors Explaining the Growing Use of Medical Assistance in Dying in Québec: Protocol for an Interdisciplinary Mixed Methods and Multimethods Study
Source: JMIR Res Protoc. 2026 Apr 20;15:e83549. doi: 10.2196/83549 (PMC13139836; doi:10.2196/83549)
Supplement: Multimedia Appendix 3 [file resprot_v15i1e83549_app3.pdf]

Fonds de recherche
Société et culture
Québec

RAPPORT D'ÉVALUATION

PROJET DE RECHERCHE / ACTIONS CONCERTÉES / MIEUX COMPRENDRE LE RECOURS À L'AIDE MÉDICALE À MOURIR EN CONTEXTE QUÉBÉCOIS – 2024-2025

INFORMATIONS SUR LE DOSSIER	
**Numéro du dossier :**	355830
**Titre :**	Mieux comprendre le recours à l'aide médicale à mourir en contexte québécois
**Nom de la personne candidate :**	Bouthillier, Marie-Eve
**Établissement :**	Université de Montréal
**Concours :**	0AMMR : 2024-2025
**Comité :**	AC-AMM-Scientifique (# 25034)
**Rôle de la personne évaluatrice :**	Interne 1

COMMENTAIRES DE LA PERSONNE ÉVALUATRICE

Projet

- Originalité et contribution à l'avancement des connaissances dans le domaine
- Clarté de la problématique, pertinence de l'approche théorique et précision des objectifs poursuivis
- Pertinence, rigueur et justification de l’approche méthodologique
- Réalisme des prévisions budgétaires et du calendrier

Parmi les points forts du projet qui ont été soulevés:

- Quelques éléments de problématiques présentés au début de la demande, et dans la section 4 chacune des sous-sections débute par une mise en contexte, ce qui permet de faire le lien avec les objectifs et de justifier le choix des méthodologies privilégiées. La demande est bien structurée.
- L’approche théorique est pertinente en regard des objectifs de la recherche.
- La démarche méthodologique est bien expliquée dans les différents volets du projet (recrutement, échantillonnage, taille des groupes, nombre d'entrevues, durée des entretiens, outils utilisés, déroulement, analyse, etc.).
- Utilisation d’une variété de méthodologies, ce qui permet une triangulation méthodologique qui favorise la fiabilité des résultats.
- La démarche utilisée pour atteindre l'objectif 3 de la section 4.4 permet l'intégration des résultats; dans cette même section la façon dont le modèle écologique de Bronfenbrenner va guider l'interprétation des résultats est expliquée.
- La programmation de recherche permet de répondre à l'ensemble des besoins de recherche spécifiques des axes 1 et 2, et également à ceux de l'axe 3.
- Considérant l’organisation du projet (différents volets répartis entre différentes sous-équipes, chacune reliée à un BRS), ajouté au nombre de chercheuses et chercheurs impliqués dans chacune des équipes, je suis d'avis que l’échéancier proposé est réaliste.

Voici quelques commentaires plus spécifiques pour chacune des sous-sections, en souhaitant que cela pourra enrichir les réflexions de l’équipe de recherche en regard de certains aspects du projet:

Dans la première section, un commentaire en lien avec le passage suivant : « Le microsystème est caractérisé par l’environnement proche de la personne. […] Mieux comprendre ce qui caractérise les individus qui ont recours à l'AMM en fonction de la perspective des proches et des soignants permettra d'apporter des éléments explicatifs complémentaires concernant le BRS 2.2.» (p. 2) Le BRS 2.2 réfère aux caractéristiques personnelles. Le lien entre les caractéristiques des individus et le microsystème ici est discutable. Les caractéristiques des individus qui ont recours à l’AMM, même si cela est en fonction de la perspective des proches et des soignants, demeurent les caractéristiques des individus (ontosystème). L’analyse du microsystème peut aider toutefois à mieux comprendre l’influence des proches, la famille par exemple, sur la prise de décision de demander, ou pas, l’AMM. Et même sur l’évolution de la santé et maladie, j’y reviens dans un autre commentaire plus loin.

Commentaire en lien avec la section 3 - Les méthodes transversales : Il est indiqué qu’il s’agit d'un devis mixte convergent. Mais lorsqu’il est question des groupes de discussions, on note que « Le guide d’animation sera construit […[à partir des informations extraites de la synthèse systématique (c.f. 3.1) et des entretiens avec les informateurs-clés (c.f. 3.2). » (extrait tiré de la demande, p. 4) Dans ce cas, alors que les résultats de deux analyses précédentes détermineront le contenu du guide d’animation, qui sera donc utilisé à une étape ultérieure, ne parle-t-on pas plutôt d’un devis mixte séquentiel ? Ou d’une double stratégie (convergent et séquentiel)?

Section 4 : Description des méthodes spécifiques par BRS

Commentaire en lien avec la section 4.1- Volet du projet spécifiquement en lien avec le BRS 2.2. On peut lire dans cette section: « Par ailleurs, ces objectifs mènent à envisager trois méthodes complémentaires de collecte de données qualitatives, comprenant des entretiens individuels, des entretiens « triadiques » et un projet pilote d’étude de dossiers. » (p. 5).

COMMENTAIRES DE LA PERSONNE ÉVALUATRICE

- En lien avec les entretiens individuels : Est-ce que le récit qui sera réalisé sera un résultat seulement? Ou servira-t-il également de corpus à partir duquel une analyse thématique sera réalisée? Est-ce qu'une analyse transversale sera réalisée?

- En lien avec l'implication « d'un de ses proches » : Comment il a été décidé (ou il sera décidé) quelles sont les personnes qui sont considérées comme des proches? Donc les personnes proches (microsystème) à inclure dans l'étude? Est-ce que ces personnes seront choisies par les chercheuses ou les chercheurs ou bien par la personne patiente (lorsque c'est possible)? Est-ce "les proches" veut dire les membres de la famille? Voici ce qui explique ces questionnements: Souvent les personnes considérées comme des « proches » par le personnel soignant sont celles qui font partie de la famille nucléaire. Or, ce n'est pas toujours le cas. Les personnes ont parfois une autre définition de la famille, et les personnes proches peuvent ne pas faire partie de la famille nucléaire. Inversement, les membres de la famille nucléaire peuvent ne pas faire partie de la vie de la personne. Je pense que le concept de « personnes proches » ou « famille » devrait être précisé en amont, ainsi que la façon dont les chercheuses et chercheurs procéderont pour identifier les personnes significatives à inclure dans l'étude. Également, en référence à l'approche théorique retenue, est-il prévu d'étudier l'impact (facteur de protection ou facteur de vulnérabilité) potentiel de la dynamique familial sur le choix de demander, ou pas, le recours à l'AMM? On sait que la santé et la maladie ont un impact sur la dynamique familiale, et qu'à l'inverse la dynamique familiale a un impact sur l'évolution de la santé et de la maladie. Dans ce contexte, explorer davantage le système familial (la dynamique, la nature des relations, la proximité ou l'éloignement des personnes significatives, les sources de stress, les conflits, les sources de soutien de différentes natures, etc.) m'apparaît important dès lors qu'on s'intéresse à l'expérience d'une personne, telle que choisir, ou pas, de demander l'AMM.

Compétences

- Qualité des expériences et des réalisations de l'équipe (activités de transfert, communications, colloques, encadrement d'étudiantes et d'étudiants, publications, subventions, etc.)
- Démonstration de l'arrimage entre l'expertise présente au sein de l'équipe et le projet

Parmi les points forts :

- La somme des expériences et des réalisations de la chercheuse principale, des 32 cochercheuses et cochercheurs et des nombreux collaborateurs impliqués dans ce projet est impressionnante.
- Toutes les expertises nécessaires pour mener un projet d'une telle envergure sont présentes dans l'équipe, tant sur le plan des méthodologies de recherche choisies que sur le plan des différents champs disciplinaires impliqués dans l'AMM.

Retombées anticipées et stratégie de mobilisation des connaissances

- Démonstration du potentiel et de la portée des résultats attendus pour l'intervention, la gestion et la prise de décision
- Stratégie de mobilisation des connaissances (moyens proposés, publics ciblés — académiques, utilisateurs et utilisatrices — incluant les partenaires de l'Action concertée, etc.)
- Liens avec les partenaires du milieu

Parmi les points forts:

- Formation d'un comité de mobilisation des connaissances
- Collaboration avec les différents partenaires pour identifier les besoins et les stratégies
- Création de trousses d'informations thématiques
- Quantité et diversité des moyens prévus

Formation

COMMENTAIRES DE LA PERSONNE ÉVALUATRICE
• Diversité des activités proposées dans le projet pour former la relève étudiante à la recherche, et variété des tâches et des responsabilités qui lui seront confiées
Parmi les points forts :<br><br>- Dix personnes étudiantes des cycles supérieurs et personne postdoctorant impliquées directement dans le projet<br>- L’équipe regroupe une quantité importante de chercheuses et chercheurs qui accompagnent plusieurs personnes étudiantes qui seront impliquées dans le projet (comme bénéficiaire d’une bourse ou comme auxiliaire de recherche).<br>- La participations d'une personnes étudiantes est indiquée dans certaines parties du projet.

RAPPORT D'ÉVALUATION

PROJET DE RECHERCHE / ACTIONS CONCERTÉES / MIEUX COMPRENDRE LE RECOURS À L'AIDE MÉDICALE À MOURIR EN CONTEXTE QUÉBÉCOIS – 2024-2025

INFORMATIONS SUR LE DOSSIER	
**Numéro du dossier :**	355830
**Titre :**	Mieux comprendre le recours à l'aide médicale à mourir en contexte québécois
**Nom de la personne candidate :**	Bouthillier, Marie-Eve
**Établissement :**	Université de Montréal
**Concours :**	0AMMR : 2024-2025
**Comité :**	AC-AMM-Scientifique (# 25034)
**Rôle de la personne évaluatrice :**	Interne 1

COMMENTAIRES DE LA PERSONNE ÉVALUATRICE
**Projet**<br>• Originalité et contribution à l'avancement des connaissances dans le domaine<br>• Clarté de la problématique, pertinence de l'approche théorique et précision des objectifs poursuivis<br>• Pertinence, rigueur et justification de l'approche méthodologique<br>• Réalisme des prévisions budgétaires et du calendrier

Points positifs

- La gouvernance du projet : comité exécutif, comité directeur, comité consultatif
- Présence d'un modèle (Bronfenbrenner) pour démêler les niveaux de détermination et donc d'explication des choix (dimension temporelle ajoutée)
- Autres sources de financement déjà acquises pour certains aspects et déjà en cours de recherche
- présence d'un système de synthèse systématique (Il ne m'est pas clair, toutefois, ce qu'on va synthétiser, s'il s'agit d'études déjà publiées ou bien de celles-là plus les résultats des études proposées, mais l'idée de faire le point des connaissances actuelles et de leur statut cognitif est clairement présente).
- Conception des études séparées tenant à l'œil les objectifs de l'appel aux propositions, spécialement la description et l'augmentation des demandes d'AMM.
- L'approche comparative systématique des études et leur organisation en profondeur visant à détecter et l'expérience vécue et – très important – la combinaisons des vues des participants dans les décisions d'AMM, et cela par différents canaux de recherche complémentaires (p.e. 18 groupes de discussion avec professionnels (projet sous 3.4), études approfondies de cas individuels, reconstruction des trajectoires de demandes, 30 GIS étudiés, …)
- Le projet sous 4.1 portant sur le BRS 2.2. : part de l'état actuel des connaissances et est innovateur. Un point vraiment fort est la méthode triple intégrant patients, leur proches, et les soignants), prolongeant une étude déjà entamée avec des membres de l'équipe au Pays Bas. L'idée de construire des trajets et leurs aboutissements des demandes est forte.
- Le sous-projet portant sur le BRS 2.3 (point 4.2 du projet de recherche) sur les modes de fonctionnement des GIS se situe (à mon avis) au centre de l'explication cherchée. Les chercheurs écrivent à juste titre le suivant : « Cette méthode a l'avantage de faciliter la description fine de systèmes complexes par les porteurs d'enjeux ainsi que de produire une évaluation rigoureuse des impacts concrets d'une intervention, d'une politique ou d'un programme et de leurs variations locales".

- Le mode de travail permet de générer des hypothèses sur l'augmentation des demandes d'AMM. L'étude proposée sous 3.2. en complément de la synthèse est très intéressante. Le choix pour l'approche internationale et comparative est bon.
- Les propositions partent de l'état actuel de connaissances et cherchent à combler les lacunes; elles formulent ici et là déjà des hypothèses qui me semblent pertinentes.
- La technique du forum communautaire est un autre point positif. C'est une forme de « citizen science » et cela pourrait générer des hypothèses intéressantes.

- En général : une approche méthodologiquement comparative (ce qui est indispensable vu l'objet du programme) (comme demandée sous Axe 1)

- Le projet de recherche couvre tous les points mentionnés dans l'appel (bien que je n'ai rien vu sur l'approche intersectionnelle, ce qui d'ailleurs me paraît secondaire dans ce genre de recherches)

- La partie juridique (BRS 1.1.) part d'hypothèses intéressantes : que la région entre droit du patient et permission légale à un médecin d'utiliser ou de prescrire un médicament léthal joue un role, importance des politiques publiques comme l'accès aux soins palliatifs, … Il aurait été intéressant d'étudier la réception différentielle des même lois dans différentes régions, de différentes lois dans un même pays (l'Australie en occurrence), et/ou la

COMMENTAIRES DE LA PERSONNE ÉVALUATRICE

dynamique de changements légaux après l'introduction de l'AMM (comme le Canada, les autres pays ont connu une pression constante sur la législation après l'introduction de la première loi autorisant EUT et/ou PAS).

- L'équipe me semble à la hauteur des résultats dans la recherche internationale sur les décisions en fin de vie

- La partie sur le BRS 1.2. (les demandes anticipées) est excellente et décrit bien les enjeux. Le cas le plus intéressant est en effet celui des Pays Bas, ou une demande anticipée est recevable mais rarement suivie, non à cause de problèmes avec la validité des requêtes mais de la réticence des médecins. Cela pourrait en effet être autre en des pays avec une culture médicale de déclarations anticipées.

- Points négatifs
- L'enquête populationnelle est conçue comparativement : c'est excellent. La méthodologie me paraît à développer. Cinq questions ici sur le juridique : (1) la connaissance légale sur n'importe quel sujet est généralement assez limitée : je me demande s'il ne faudrait pas inclure les répondants sur base de leur probabilité de s'être informés sur l'AMM, les soins palliatifs, etc.; (2) deux des juridictions inclues (Belgique et Suisse) sont multi-linguistiques et les taux de PAS ou d'euthanasie (les lois demeurant les mêmes) sont différents selon les communautés linguistiques – impossible de les ajouter par nationalité faute d'éliminer des différences très réelles; (3) pondération, oui, mais pas facile d'obtenir partout les données requises; (4) quant à l'évolution des taux de PAS et EUT : dans certains pays leur nombre est repérable, dans d'autres pas (le nombre rapporté aux autorités peut être différent du nombre pratiqué); (5) il faudrait prendre en compte l'évolution des alternatives pour EUT et PAS (plus particulièrement la sédation profonde continue, - croissance spectaculaire dans certains pays, notamment aux Pays-Bas et en Suisse, allant de pair avec un accroissement de EUT et/ou PAS)
- On aurait pu partir d'hypothèses dérivées d'autres études. Je donne quelques exemples : au Pays-Bas le taux d'euthanasie a baissé en 2018. L' explication en est probablement la lassitude d'euthanasie chez les médecins et la substitution de la sédation continue à l'euthanasie. On aurait donc pu partir de la question de la substituabilité des pratiques à la fin de la vie, et ne pas s'en tenir à l'AMM. En Suisse, l'augmentation de PAS est le résultat d'un facteur démographique et non d'une culture légistique: le vieillissement de la population. Nous avons constaté que le progrès de l'euthanasie dans le région bruxelloise est dû à la scolarisation croissante des +60. L'effet du facteur linguistique a été démontré et il est probable qu'une même loi (en Europe) est influencé par la culture linquistique médicale (la Suisse allemande ressemblant plus l'Allemagne et la Belgique francophone la France). On a trouvé aussi que le taux d'euthanasie est très sensible au contrôle réel judiciaire. Et ainsi de suite.

- Le volet sociétal part d'hypothèses (cela pourrait facilement tenir de la « fishing expedition »), n'apporte pas de grandes surprises, mais est bien - que ce soit schématiquement - élaboré. Internationalement, il y a peu de variabilité dans les facteurs de base, s'agissant de régimes politiques libéraux. Un point fort ici est la collaboration étroite avec l'équipe 2.3 qui est orienté sur les facteurs de différentiation.
- La revue des médias n'a rien de surprenant non plus. Il faudrait étudier l'impact de publications périodiques sur des communautés spécifiques.
- Il m'est difficile de me faire une idée du réalisme des prévisions budgétaires. Quant au calendrier : il est très serré, mais vu l'ampleur de l'équipe – réalisable.

Compétences

- Qualité des expériences et des réalisations de l'équipe (activités de transfert, communications, colloques, encadrement d'étudiantes et d'étudiants, publications, subventions, etc.)

COMMENTAIRES DE LA PERSONNE ÉVALUATRICE
• Démonstration de l'arrimage entre l'expertise présente au sein de l'équipe et le projet
Rien à remarquer : l’équipe réunit une grande variété de compétences pertinentes et nécessaires, et bien plus, pour réaliser les recherches et les autres activités. L’équipe a déjà une grande expérience, très variée par ailleurs, dans le domaine de l’étude de la fin de la vie (dans le domaine médical).
**Retombées anticipées et stratégie de mobilisation des connaissances**
• Démonstration du potentiel et de la portée des résultats attendus pour l'intervention, la gestion et la prise de décision<br>• Stratégie de mobilisation des connaissances (moyens proposés, publics ciblés — académiques, utilisateurs et utilisatrices — incluant les partenaires de l'Action concertée, etc.)<br>• Liens avec les partenaires du milieu
La liste des publics ciblés et moyens utilisés est impressionante. On voit aussi dans les budgets qu’on a pensé à inclure des spécialistes dans le domaine des transferts de la connaissance. Voir aussi le comité de mobilisation. Le grand public, les associations pour le droit de mourir en dignité, les associations de patients ne sont pas mentionnés. C'est regrettable - mais à décharge: il y a une composante citizen science dans le projet.
**Formation**
• Diversité des activités proposées dans le projet pour former la relève étudiante à la recherche, et variété des tâches et des responsabilités qui lui seront confiées
Activités très variées, nombre d'étudiants-chercheurs bien détaillé et justifié.

Fonds de recherche
Société et culture
Québec

RAPPORT D'ÉVALUATION

PROJET DE RECHERCHE / ACTIONS CONCERTÉES / MIEUX COMPRENDRE LE RECOURS À L'AIDE MÉDICALE À MOURIR EN CONTEXTE QUÉBÉCOIS – 2024-2025

INFORMATIONS SUR LE DOSSIER	
**Numéro du dossier :**	355830
**Titre :**	Mieux comprendre le recours à l'aide médicale à mourir en contexte québécois
**Nom de la personne candidate :**	Bouthillier, Marie-Eve
**Établissement :**	Université de Montréal
**Concours :**	0AMMR : 2024-2025
**Comité :**	AC-AMM-Scientifique (# 25034)
**Rôle de la personne évaluatrice :**	Interne 1

COMMENTAIRES DE LA PERSONNE ÉVALUATRICE

Projet

- Originalité et contribution à l'avancement des connaissances dans le domaine
- Clarté de la problématique, pertinence de l'approche théorique et précision des objectifs poursuivis
- Pertinence, rigueur et justification de l’approche méthodologique
- Réalisme des prévisions budgétaires et du calendrier

Le projet est mature, important et expose scientifiquement et méthodiquement les étapes qu'il entend suivre pour répondre aux objectifs demandés dans l'appel à projet.
Il manifeste sans aucun doute un investissement réel afin d'apporter des réponses circonstanciées et scientifiquement adaptées aux attentes de l'appel à propositions lancé.
Il a recours à diverses méthodes pertinemment adaptées aux attentes de l'appel. Parmi les difficultés on relèvera des enquêtes populationnelles vastes à mener, comparatives et auprès de publics différents: les personnes demandant l'AMM, leurs proches, les soignants et intervenants dans leurs expériences, l'acceptabilité sociale, les institutions...
La diversité de ces publics, de ces approches et de ces analyses (parfaitement logiques et nécessaires d'un point de vue scientifique) implique une structuration minutieuse de l'approche et une méthode anticipée d'identification et de traitement des données qui apparaissent ici pleinement réfléchies.
à des "méthodes transversales, composées d’une synthèse systématique des écrits, d’entretiens avec des informateurs clés, d’une enquête populationnelle et de groupes de discussions avec des professionnels impliqués dans l’AMM seront utilisées pour saisir les facteurs explicatifs de l’évolution du recours à l’AMM. La synthèse systématique couvrira l’ensemble des pays où une aide active à mourir est permise, alors que les trois autres méthodes permettront de sonder spécifiquement volet sociétal des entretiens auprès de personnes ayant formulé une demande d’AMM, leurs proches et leurs soignants. Ce volet sera complété par un projet pilote d’étude de dossiers. Pour mettre en lumière les
différences dans l’organisation des soins et services entre les régions, une base de données sera
créée, ce qui nous permettra de faire un portrait des facteurs explicatifs (modèle de system mappping).
Le volet sociétal sera étudié à l’aide de deux méthodes : une revue des textes médiatiques québécois
et des forums communautaires pour consulter la population de diverses régions du Québec. Pour analyser et comparer les politiques publiques, une synthèse réaliste et une analyse juridique positiviste
seront réalisées. Cette étape nous permettra d’intégrer les résultats de notre projet".
La pleine adéquation des méthodes envisagées et des objets d'analyses exposés dans le dossier se fait avec une telle précision que la lecture d'ensemble noie un peu les objectifs globaux, parfois difficiles à identifier au sein de ce dossier volumineux et parfois presque trop "administratif".
Les prévisions budgétaires sont précises et circonstanciées, surveillant notamment les proportionnalités entre intervenants selon leurs domaines ou leur ancienneté, mais en cherchant à doter de façon plus que subséquente les étudiants ou jeunes chercheurs.

Compétences

- Qualité des expériences et des réalisations de l'équipe (activités de transfert, communications, colloques, encadrement d'étudiantes et d'étudiants, publications, subventions, etc.)
- Démonstration de l'arrimage entre l'expertise présente au sein de l'équipe et le projet

De nombreuses subventions préexistantes déjà obtenues (près d'une dizaine) témoignent d'un savoir faire, d'un faire savoir mais surtout d'une anticipation effective et concrète du projet.
L'équipe comporte un nombre important de membres (75) mais leur organisation est réfléchie et structurée dans une coordination qui apparaît effective et réaliste pour que le nombre élevé ne devienne pas un poids. Au regard des attentes de l'appel à propositions, néanmoins, l'importance de l'équipe doit être vue comme un gage de réalisme afin de pouvoir mener à bien la tâche envisagée.
Les curriculums vitae transmis confirment la diversité et la pertinence de la composition de l'équipe : universitaires, cliniciens, juristes, éthique, soins palliatifs, psychologie, soins infirmiers, neurologie, santé, gériatrie...

COMMENTAIRES DE LA PERSONNE ÉVALUATRICE
Les chercheurs sont issus d'universités diverses (Montreal, Sherbrooke, Laval, MacGill) mais également international (en considération des pays ayant accueilli l'aide à mourir : Bruxelles, Lausanne, Rotterdam) même si sur ce dernier point les contacts des quelques collaborateurs issus de pays extérieurs au Québec apparaissent très limités (alors que l'appel à propositions invitait à un regard comparatif avec des pays extérieurs ayant également recours à l'aide médicale à mourir).

Retombées anticipées et stratégie de mobilisation des connaissances

- Démonstration du potentiel et de la portée des résultats attendus pour l'intervention, la gestion et la prise de décision
- Stratégie de mobilisation des connaissances (moyens proposés, publics ciblés — académiques, utilisateurs et utilisatrices — incluant les partenaires de l'Action concertée, etc.)
- Liens avec les partenaires du milieu

Les publics ciblés sont judicieusement réfléchis et mobilisés.
dans le cadre de l'organisation de la gouvernance (méthodiquement élaborée au travers de) est institué un comité spécialisé en mobilisation des connaissances auxquels différents bénéficiaires seront
invités à participer (représentants des instances décisionnelles, professionnelles, groupes et associations).
La gouvernance est pensée de façon opérationnelle : un comité exécutif3 responsable de l'opérationnalisation et de la
coordination générale du CIRAMM (rencontres quotidiennes). Un comité directeur composé de cliniciens, chercheurs et patiente partenaire soutient le comité exécutif dans les décisions et orientations générales (rencontres mensuelles). Un comité scientifique réunit l'ensemble des responsables des cinq BRS4 (rencontres semestrielles), dont les sous-équipes se réuniront en fonction de l'avancement des projets et
échéanciers, un comité consultatif international
Par ailleurs, au regard de la notoriété légitime du pilote du projet Consortium interdisciplinaire de recherche sur l'aide médicale à mourir (CIRAMM), les résultats attendus auront vocation à inspirer les cliniciens et les organisations dans les bonnes pratiques à implanter. De même, les stratégies de mobilisation et de transfert des connaissances susceptibles de générer des idées innovantes visant le déploiement d'une pratique éthique des soins de fin de vie au Québec.
De nombreux partenaires sont choisis notamment dans un objectif de mobilisation des connaissances et de diffusion des résultats attendus, diffusion de l'information aux différentes étapes de la recherche (sondages, entretiens, groupes de discussion, forums citoyens) pour
comprendre l'impact de l'évolution des demandes au Québec, les distinctions dans l'offre de service en matière de soins de fin de vie et les disparités régionales.
Les liens avec les partenaires du milieu sont plus que notables. le projet bénéficie non seulement d'une expérience construite en amont par des chercheurs de compétence et d'expérience manifestes, mais le dossier traduit aussi un investissement concret dans les attentes de l'appel à propositions lancé.
Parmi les lettres de soutien on retrouve ainsi : l'université de Montreal, le centre intégré de santé et de services sociaux de Laval, Groupes interdisciplinaires de soutien (GIS)
du réseau de la santé et des services sociaux, le Réseau québécois de recherche en soins palliatifs et de fin de vie (RQSPAL), l'association canadienne des évaluateurs et prestataires de l'AMM (ACEPA), Communauté de pratique AMM-Québec (CPAQ), la FÉDÉRATION DES MÉDECINS OMNIPRATICIENS DU QUÉBEC...

Formation

- Diversité des activités proposées dans le projet pour former la relève étudiante à la recherche, et variété des tâches et des responsabilités qui lui seront confiées

Des étudiants sont clairement et expressément associés à la recherche en tant que membres actifs. PLusieurs individualités sont d'ores et déjà identifiées dès cette première étape de candidature, ce qui augure positivement de la réalité de la poursuite de la formation de la relève étudiante dans les mois et années à suivre du projet.
Sur une équipe constituée de 75 personnes, le dossier fait état de la répartition suivante en matière de recherche :

COMMENTAIRES DE LA PERSONNE ÉVALUATRICE

"33 chercheurs (statut 1 et 2) et 10 étudiants des cycles supérieurs et postdoctorants de huit universités québécoises et d'un centre médical universitaire néerlandais (UdeM, ULaval, USherbrooke, UQAM, UMcGill, UQTR, UQAR, UQAC, Radboudumc)". Diversité, niveau, nombre et proporotions apparaissent dès lors pleinement satisfaisants.

Les dotations du budget prévisionnel intègrent de façon significative et organisée le soutien aux étudiants :

"Dès la création de notre consortium, dix étudiants ont été intégrés et certains ont même été désignés co-porteurs de BRS (postdoctorantes Girard (2.2) et Plaisance (1.1)). Sur les trois ans, le CIRAMM financera : une bourse de premier cycle, quatre bourses de maîtrise, une bourse de doctorat et deux bourses postdoctorales, sans compter une bourse postdoctorale supplémentaire assumée conjointement par le CIRAMM et l'une de ses collaboratrices (Marcoux). Nous offrirons aussi quatre assistanats de recherche de 2e cycle et six de 3e cycle."

Le vivier d'étudiants est issu de divers parcours, mais logiquement un certain nombre est en lien (ou l'a été) avec le Réseau québécois de recherche en soins palliatifs et de fin de vie (RQSPAL).

Des rôles dédiés leur sont attribués, en coordination avec les équipes et membres seniors.

Ainsi pour au sein des besoins de recherche spécifiques mentionnés par le projet, le dossier fait-il état pour le BRS 2.2 consacré aux caractéristiques des personnes demandant ou ayant demandé le recours à l'aide médicale à mourir est-il prévu que "Accompagnée d'un étudiant, la chercheure responsable révisera chaque dossier de façon à documenter la trajectoire de la demande d'AMM (administrée, refusée, retirée, décès survenu avant l'AMM), ainsi que les propos des demandeurs et les explications des professionnels."

De même dans les besoins de recherches spécifiques 2.1 (les candidats ont reformulé les BRS) consacré à l'objectif d'identification des facteurs sociétaux et juridiques potentiellement explicatifs de l'augmentation du recours à l'AMM au Québec est-il prévu qu'à l'issue de la collecte des données la sélection des textes sera effectuée par "deux personnes (un étudiant et un chercheur)".

La volonté d'association des étudiants constitue une réalité au sein du projet qui s'appuie de façon opérationnelle et formatrice sur ces compétences :

"Les étudiants participeront à toutes les étapes de réalisation du projet : élaboration des outils, collectes, analyses, interprétations des données et leur restitution sous la forme de produits scientifiques variés (articles scientifiques, communications orales et affiches, notes politiques, rapports de recherche). Avec leur apport, nous serons en mesure de réaliser des balados et autres activités de vulgarisation scientifique pour le grand public. Les étudiants affiliés au CIRAMM seront encouragés à participer à différentes activités scientifiques. Entre autres, nous les inviterons à faire partie du comité étudiants du RQSPAL. Ils pourront ainsi s'impliquer dans l'organisation des journées scientifiques étudiantes et participer à des activités de retraite d'écriture organisées de concert avec l'organisme Thèsez-vous. Ils pourront assister à de multiples activités de formation (par ex.: sur la réalisation de synthèses systématiques, les analyses qualitatives, la rédaction de demandes de subvention) et de midis conférences scientifiques. Enfin, nos membres valoriseront la participation des étudiants du CIRAMM à diverses activités de réseautage, en les intégrant à leurs réseaux scientifiques, cliniques et communautaires. Ainsi, nous contribuerons au développement de leurs savoirs, de leur sensibilité, de leurs compétences méthodologiques et humaines nécessaires à la recherche dans le domaine des SPFV. Nous comptons ainsi contribuer à la pérennité et au dynamisme dans la recherche associée à l'AMM et aux SPFV au Québec."
